# The Potential of Visible Spectroscopy as a Tool for the In-Line Monitoring of Lignin Methylolation

**DOI:** 10.3390/polym15010178

**Published:** 2022-12-30

**Authors:** Sofia Gonçalves, Jorge Martins, Nádia T. Paiva, Diana Paiva, Luísa H. Carvalho, Fernão D. Magalhães

**Affiliations:** 1 LEPABE—Faculdade de Engenharia da Universidade do Porto, Rua Dr. Roberto Frias, 4200-465 Porto, Portugal; 2 Associate Laboratory in Chemical Engineering (ALiCE), FEUP, 4200-465 Porto, Portugal; 3 DEMad–Departamento de Engenharia de Madeiras, Instituto Politécnico de Viseu, Campus Politécnico de Repeses, 3504-510 Viseu, Portugal; 4 Sonae Arauco Portugal S.A., Lugar do Espido—Via Norte, 4470-177 Porto, Portugal

**Keywords:** lignin, methylolation, spectroscopy

## Abstract

Out of the 50 to 70 million tons of lignin that are produced annually, only 1 to 2% are used for value-added products. Currently, 90% of the total market of this compound corresponds to lignosulphonates (LS). The most successful industrial attempts to use lignin for wood adhesives rely on using it as a partial substitute in phenol–formaldehyde or urea–formaldehyde resins. However, lignin’s aromatic ring presents a low number of reactive sites. Several methods have been proposed to improve its reactivity, such as prior methylolation with formaldehyde. Off-line methods are commonly applied to monitor this reaction’s progress, but this introduces a significant delay in the analysis. This study proposes a new method for in-line monitoring of the methylolation reaction using visible spectroscopy. In order to monitor the reaction progress, principal component analysis was applied to the spectra, and the obtained scores were analyzed. When these results were plotted against those obtained by the off-line methods, a satisfactory regression was obtained at 50 °C (R^2^ = 0.97) and 60 °C (R^2^ = 0.98) for two different LS samples. Therefore, it was concluded that visible spectroscopy is a promising technique for studying lignin methylolation.

## 1. Introduction

As the most abundant renewable source of phenolic compounds of natural origin, lignin has been increasingly researched as an eco-friendly alternative to petroleum-based compounds [1].

The large amounts of lignin waste have been proposed for adhesive production, since the beginning of the wood pulping industry. Particularly for lignin-based wood adhesives, many studies have been reported. The most successful industrial attempts comprised using lignin in combination with phenol–formaldehyde (PF) resins or urea–formaldehyde (UF) resins [2,3,4]. However, no-added-formaldehyde systems have also been proposed that combine lignin with polyethylenimine, furfuryl alcohol, polymeric isocyanate, chitosan, tannins, glyoxal, wheat flour, poly(vinyl alcohol), and hexamine [3,5,6,7,8,9,10,11].

Lignin comprises three monolignols: p-hidroxyphenyl (H), guaiacyl (G), and syringyl (S) units [1,12,13]. Different types of plants display different degrees of participation of these major monolignols. The main monolignols in softwood and hardwoods are guaiacyl and syringyl units, respectively [14].

**Figure 1:**
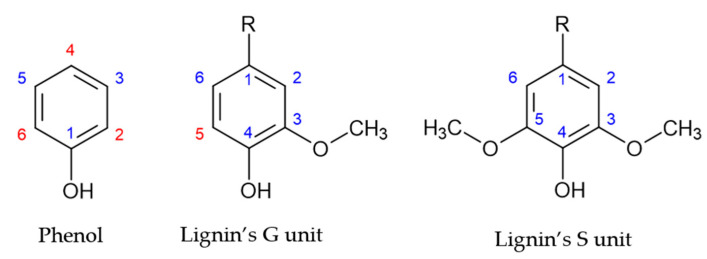
Reactive sites of phenol and lignin’s phenolic units (adapted from [2]).

Lignin’s monolignol units present a similar structure to that of phenol, as shown in Figure 1. However, the aromatic ring of lignin’s G and S units are blocked in positions 1 and 3, and 1, 3 and 5, respectively. Consequently, lignin units present a lower number of reactive sites, as shown by the red numbers in Figure 1. Therefore, the methoxyl groups are the cause of lignin’s low reactivity. This has hindered the application of lignin in resin synthesis [2,15].

However, in recent studies it was suggested that other characteristics of lignin might also affect its incorporation in resins. These include molecular branching and conformation steric availability, molar mass, and distribution. Thus, hardwood lignins may still be suitable phenol substitutes [16].

Many approaches have been suggested to increase the reactivity of lignin molecules and, consequently, facilitate their incorporation in resins. These include phenolation, hydrolysis, oxidation, treatment with ionic liquids, and, lastly, methylolation [4,15,17,18,19,20,21].

**Figure 2:**
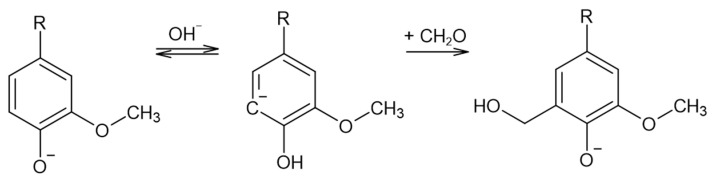
The Lederer–Manasse reaction (adapted from [4,22]).

Lignin methylolation, or hydroxymethylation, introduces hydroxymethyl groups (–CH_2_OH) onto lignin molecules. This reaction usually takes place with formaldehyde in an alkaline medium. The resultant lignin can be directly incorporated as a phenol substitute in the synthesis of phenol–formaldehyde (PF) resol resins for wood adhesives [18]. Under these conditions, the Lederer–Manasse reaction is predominant. Lignin’s reactivity is increased via the incorporation of hydroxymethyl groups into the aromatic rings, as shown in Figure 2. Undesirable secondary reactions involving the lignin molecule include the Tollens reaction and further condensations in which methylene bonds are formed. On the other hand, the Cannizzaro reaction, in which formaldehyde reacts with itself, may also take place under more extreme conditions [18,22,23].

Lignin’s reactivity depends not only on the reaction conditions but also on the lignin’s source and pulping process [22].

A limited amount of studies exist on the reaction between formaldehyde and lignin. Usually, the reaction progress is monitored through the disappearance of formaldehyde. However, other techniques have been used, such as differential scanning calorimetry (DSC), nuclear magnetic resonance spectroscopy (NMR), Fourier transform infrared spectroscopy (FTIR) and ultraviolet–visible (UV-vis) spectroscopy [23,24,25,26,27]. The reaction has been described as being a second-order reaction [24].

Peng et al. studied the methylolation of ammonium and sodium lignosulfonate by DSC, ^1^H NMR, and monitoring the free formaldehyde content. Kinetic parameters were obtained at 20, 40, 50, and 60 °C. It was concluded that the reactivity of the LS samples was influenced by the number and availability of reactive sites per C9 unit of lignin. The lignin samples also needed a shorter time, about 2 h, to reach complete methylolation at low temperatures when compared with phenol [24].

In a similar analysis, Alonso and co-workers studied the methylolation of softwood and hardwood LS samples. Through a preliminary FTIR and ^1^H NMR analysis, it was concluded that the softwood ammonium LS sample displayed the most promising characteristics. The progress of this sample‘s methylolation was followed by ^1^H NMR and the determination of the free formaldehyde content in order to optimize the respective operating conditions. The chosen conditions corresponded to a 1.0 formaldehyde-to-lignin molar ratio, 45 °C, and a sodium hydroxide-to-lignin molar ratio of 0.80. The remaining lignin samples were methylolated under these conditions. Overall, the hardwood LS samples resulted in a lower consumption of formaldehyde and therefore presented a lower reactivity. This low performance was justified by the fact that their predominant structural unit, syringyl, displays a higher degree of substitution on the aromatic ring [23].

In an older study, the methylolation of lignin model compounds in their meta positions was studied using ^1^H NMR. Both phenolic and non-phenolic softwood and hardwood model compounds were modified with formaldehyde in an acidic medium with dioxane [27].

In an innovative approach, Taverna et al. recently studied the methylolation reaction for different lignin samples by monitoring the decay in phenolic hydroxyl groups by UV–Vis spectroscopy. This method was applied simultaneously with the determination of the free formaldehyde content. This reaction was studied under different temperatures (40, 50, and 70 °C), pHs (9 and 11) and initial formaldehyde/lignin weight ratios (0.07 and 1.47). The authors were able to prove that, in the absence of secondary reactions, the consumption of formaldehyde corresponded to that of reactive aromatic hydrogens [26].

Bernar et al. studied the hydroxymethylation of acetosolv lignin from sugarcane bagasse at 40 °C, between 0.25 and 8 h, using FTIR spectroscopy and principal component analysis (PCA). Samples of the reaction mixture were purified prior to the FTIR analysis through precipitation, filtration, and drying. After assessing the scores of the first principal component, the authors concluded that the reaction ceased after 4 h. These results were confirmed by the consumption of formaldehyde, which also stopped. After this time, other variations in the FTIR spectra were attributed to the condensation of the lignin fragments, forming a resol-type crosslinked resin [25]. 

While formaldehyde consumption appears to be an adequate method for following the methylolation of lignin, it must be used with caution. This is due to the fact that formaldehyde can also react in secondary reactions, such as the Cannizaro reaction. In order to account for this, studies have included a blank trial in which the lignin is excluded from the reaction mixture. Therefore, the formaldehyde consumption in the Cannizaro reaction is accounted for [26,28]. However, it should also be noted that formaldehyde can also react with the ammonium ion present in ammonium LS [29,30].

Although these studies successfully study and monitor the methylolation reaction, they also present a common limitation: they resort to off-line methods, which are time-consuming, destructive and require sample preparation. This current work aims at developing, for the first time, an in situ in-line methodology based on visible spectroscopy to monitor lignin methylolation.

It should be noted that, in other studies, different lignin modifications were monitored successfully through in-line approaches other than visible spectroscopy. For example, lignin depolymerization was effectively monitored through the combination of attenuated total-reflectance infrared spectroscopy (FTIR-ATR) and chemometric methods, PCA and partial least squares (PLS) regression [31]. On the other hand, Cateto et al. also used FTIR-ATR to monitor the synthesis of lignin-based polyurethanes [32]. In another approach, Liu and co-workers observed the progress of the hydroxyethyl modification by measuring the CO_2_ produced in the reaction [33].

Nevertheless, to the best of our knowledge, our approach has never been applied to monitor lignin methylolation.

## 2. Materials and Methods

Thick, spent sulfite liquor (HLS) from the acidic magnesium-based sulfite pulping process of *Eucalyptus globulus* (hardwood) was supplied by Caima-Indústria de Celulose SA (Constância, Portugal). Spray-dried sodium lignosulphonate (SLS) (STARLIG^®^NA96K) from the sodium bisulfite pulping process of *Picea abies* (softwood) was supplied by LignoStar International BV (Hague, The Netherlands). Unless stated otherwise, all the other chemicals were provided by Euroresinas-Indústrias Químicas, S.A. (Sines, Portugal).

### 2.1. Physico-Chemical Characterization of the LS Samples

For the determination of the dry matter content, the samples were dried in a ventilated oven at 105 °C until a constant mass was reached [34]. The ash content of the samples was determined gravimetrically after ignition in a muffle furnace at 525 °C, according to ISO 1762 [35].

For the spent sulphite liquor sample, the pH, density, and viscosity were also determined. Further, pH values were evaluated using an InLab Routine Pro combined pH glass electrode with an integrated temperature probe (Mettler Toledo, Columbus, OH, USA). The density and viscosity determinations were conducted using a hydrometer (Ludwig Schneider, Wertheim, Germany) and a Brookfield Model DV-II + viscometer with spindle 2 at 60 rpm (AMETEK Brookfield, Middleboro, MA, USA), respectively.

In order to determine the lignosulphonate content, 0.5 g of HLS was diluted with deionized water in a 1000 mL volumetric flask. In this case, the pH was previously adjusted to 5. A similar solution was prepared for the SLS, taking into account the solids content of the HLS. Then, 0.5 mL of these solutions was transferred to a quartz cuvette and diluted to 3.0 mL with deionized water [36]. The LS content of the samples was calculated from the absorbance at a wavelength of 232.5 nm, considering an extinction coefficient of 24.5 L g^−1^ cm^−1^ [23,37]. A GENESYS 10S UV-Vis spectrophotometer (Thermo Scientific, Waltham, MA, USA) was used.

#### 2.1.1. Content of Phenolic Hydroxyl Groups

In order to determine the content of the phenolic hydroxyl groups, the lignosulphonate samples were weighed into a 50 mL volumetric flask and diluted with deionized water. Then, 3.0 mL of this solution was transferred to three 25 mL volumetric flasks and diluted using a buffer solution with a pH of 6 (reference) or 12 or an aqueous NaOH 0.2 M solution. The final lignosulphonate concentration of these solutions was about 0.08 g/L. The spectra of these solutions were recorded in the range of 190 to 400 nm [38,39]. A GENESYS 10S UV-Vis spectrophotometer (Thermo Scientific, Waltham, MA, USA) was used.

**Figure 3:**
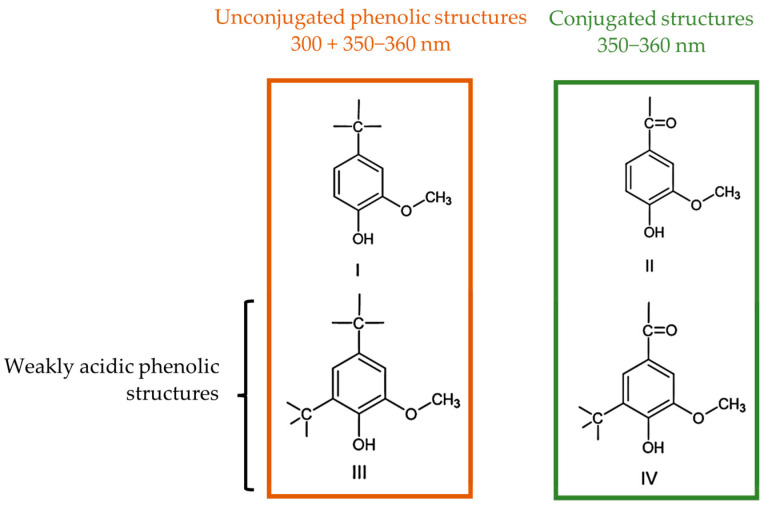
Phenolic structures identified by the UV method (adapted from [38,39]).

**Figure 4:**
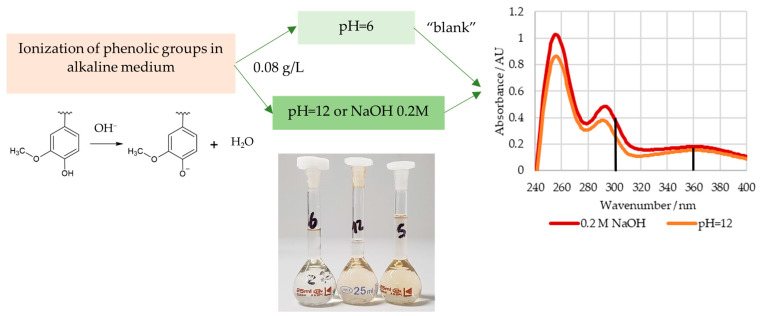
Procedure for the determination of the content of phenolic hydroxyl groups.

The ionization spectra of the analyzed LS samples were found to have two maxima near 300 and 360 nm, which are associated with the structures shown in Figure 3 [39]. The content of weakly acidic types III and IV, ${\left[OH\right]}_{III+IV}$, types I and II, ${\left[OH\right]}_{I+II}$, and the total, ${\left[OH\right]}_{I+II+III+IV}$, phenolic hydroxyl groups were then determined according to Zakis et al. This was carried out considering that the phenolic groups, type I and II, are readily ionized in the alkaline pH 12 solution, whilst the remaining groups require the higher pH of the NaOH 0.2 M solution [39]. A concise scheme with the procedure for this method is shown in Figure 4.

#### 2.1.2. Fourier Transform Infrared Spectroscopy (FTIR)

The infrared spectra were recorded using a VERTEX 70 FTIR spectrometer (Bruker, MA, USA) in the transmittance mode with a high-sensitivity DLaTGS detector at room temperature. The samples were measured in ATR mode, with an A225/Q PLATINUM ATR Diamond crystal with a single reflection accessory. The spectra were recorded from 4000 to 500 cm^−1^ with a resolution of 4 cm^−1^. The samples were previously dried at 105 °C.

#### 2.1.3. Methylolation of LS

Firstly, the jacketed reactor was loaded with the LS samples, and deionized water was added to adjust the final concentration of lignosulphonate in the reaction medium to approximately 280 g/L [23,28,40,41]. This concentration was selected as it minimized the formation of agglomerates in the reaction medium [28,42]. Then, the pH was adjusted to 9.6 using a NaOH solution at 50% (*w*/*w*). Lastly, formaldehyde 50% (*w*/*w*) was added to a formaldehyde-to-LS solids ratio of 0.17 at the selected reaction temperature. The mixture was left to react for 5 h [23,28,40,41].

### 2.2. Monitoring of the Methylolation Reaction

#### 2.2.1. Phenolic Hydroxyl Groups Type I and II

The concentration of the phenolic hydroxyl group types I and II was used for the off-line monitoring of the LS methylolation. Two samples with 0.15 g of the reaction medium were taken every hour for further analysis and diluted in 50 mL volumetric flasks. The determination of the content of the phenolic hydroxyl groups was then carried out, as explained previously [26,39]. It should be noted that this analysis took at least 15 min and that it was observed that the reaction could still progress slowly in the volumetric flasks. 

**Figure 5:**
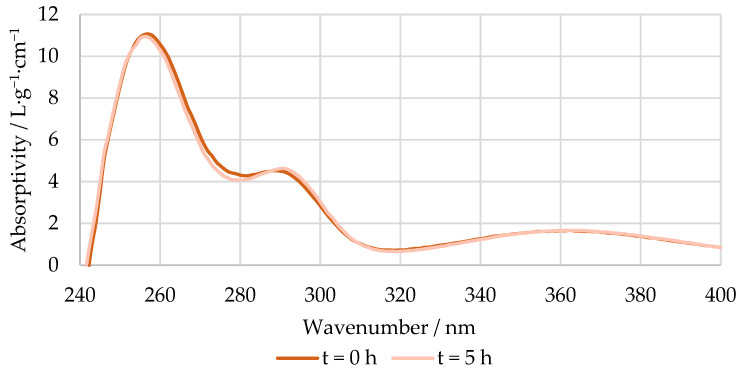
Absorptivity vs. wavenumber of the reaction mixture at pH 12 at the beginning and end of the methylolation of HLS at 50 °C.

An example of the absorptivity at a pH 12 of the reaction mixture at the beginning and end of the methylolation of HLS at 50 °C is shown in Figure 5. As mentioned previously, the spectra of the pH 6 solutions were used as blanks.

#### 2.2.2. Free Formaldehyde

The progress of the methylolation reactions was also followed through the off-line determination of the free formaldehyde content. This procedure was carried out according to ISO 11402:2004 with some minor alterations, as suggested by Mansouri et al. Thus, 3 g of the reaction mixture was weighed into a beaker. Then, 30 mL of deionized water and 20 mL of methanol were added. The free formaldehyde content was then determined through the hydroxylamine hydrochloride titration method [20,43].

#### 2.2.3. Determination of the Kinetic Parameters

The reaction between lignin and formaldehyde has been previously studied and described as a second-order reaction. In this case, where the reaction occurs as F+POH→C, the reaction rate for a selected temperature, *T*, is described by the following equation:

(1) $$-\frac{d\left[F\right]}{dt}=k_{1}\left(T\right)\cdot \left[POH\right]\cdot \left[F\right]$$

where $k_{1}\left(T\right)$ is the rate constant of the methylolation reaction, in L·mol^−1^·h^−1^, at a constant temperature, T; [F] and [POH] are the concentrations of the formaldehyde and phenolic groups type I and II in mol L^−1^, respectively, and t is the time in hours [24,44].

It was admitted that only a portion of the phenolic groups in lignin are available to react with formaldehyde. Therefore, the progress of the reaction, x, was given by the following equation:

(2) $$x={\left[F\right]}_{0}-{\left[F\right]}_{t}={\left[POH\right]}_{reactive}-{\left[POH\right]}_{t}$$

where ${\left[F\right]}_{0}$ is the initial concentration of formaldehyde; ${\left[F\right]}_{t}\;$and ${\left[POH\right]}_{t}\;$are the concentrations of the formaldehyde and phenolic groups type I and II at time t, respectively; ${\left[POH\right]}_{reactive}$ is the concentration of the phenolic groups type I and II available to react with formaldehyde at the beginning of the reaction [24,44].

Taking this into account, ${\left[POH\right]}_{reactive}$ was determined as follows:

(3) $${\left[POH\right]}_{reactive}={\left[POH\right]}_{0}\cdot \left(1-\%POH_{non-reactive}\right)$$

where ${\left[POH\right]}_{0}$ is the initial concentrations of phenolic groups type I and II and $\%POH_{non-reactive}$ is the percentage of the non-reactive phenolic groups.

For the HLS sample, a secondary reaction was also considered; thus, the rate of the disappearance of formaldehyde was given by the following equation:

(4) $$-\frac{d\left[F\right]}{dt}=k_{1}\left(T\right)\cdot \left[POH\right]\cdot \left[F\right]+k_{2}\left(T\right)\cdot {\left[F\right]}^{2}$$

where $k_{2}\left(T\right)$ is the rate constant of the secondary reaction, in L mol^−1^ h^−1^, at a constant temperature [24,44].

#### 2.2.4. Spectra Acquisition

The spectrometer used for the in-line acquisition of the visible spectra of the reaction medium was a Torus Concave Grating Spectrometer (Ocean Optics, Ettlingen, Germany) equipped with a Solvias FLEX transflection probe (Solvias, Kaiseraugst, Switzerland). For this purpose, the probe was immersed directly in the reaction mixture and spectra acquisition was commenced when formaldehyde was added. Two spectra were acquired per minute using 32 scans, a resolution of 0.24 nm, and an integration time of 1 ms. The wavelength range was from 342 to 825 nm. The software used for data collection was Ocean View 1.6 (Ocean Insight, Orlando, FL, USA).

#### 2.2.5. Principal Component Analysis

After the spectra acquisition, Principal component analysis (PCA) was carried out on the mean-centered spectra using Octave 6.4 software. PCA has been used previously to monitor chemical processes and reactions [25,45,46]. This method allowed the representation of a large amount of data in a smaller dimensional space. The data was divided into principal components orthogonal to each other, where the first component accounts for the largest amount of variance and the others successively account for less variance [45,47].

When PCA is applied to a data matrix, X, the data is divided into scores, $t_{k}$, and loadings, $p_{k}$. These provide information on how the samples, in this case, spectra, or the process variables and wavelengths, relate to each other, respectively. Hence, the mathematic equation for PCA is the following:

(5) $$X=\overset{np}{\underset{k=1}{\sum }}t_{k}\cdot p_{k}^{T}+E=T\cdot P^{T}+E$$

where np corresponds to the number of principal components; T, in superscript, is the transpose of the matrix; T is the scores matrix; P is the loadings matrix, and E is the error term, which contains information that is not included in the first PCs [45,48]. For more insights into the PCA methodology, the reader is directed to the cited references.

## 3. Results and Discussion

### 3.1. Characterization of the LS Samples

Before the production of adhesives, the available LS samples were characterized. Two samples were analyzed: thick, spent sulfite liquor (HLS) from the acidic magnesium-based sulfite pulping process of *Eucalyptus globulus* (hardwood) and spray-dried sodium lignosulphonate (SLS) from the sodium bisulfite pulping process of *Picea abies* (softwood).

The results of the physico-chemical characterization and the phenolic hydroxyl group content are shown in Table 1 and Table 2, respectively.

The results obtained in this study for HLS are consistent with those found in the literature [34,37,49,50,51].

SLS contains a higher content of lignosulphonates as well as a higher ash content. On the other hand, HLS possesses a larger amount of phenolic hydroxyl groups. Nevertheless, these are mostly syringyl units, which present a lower amount of reactive sites in their aromatic ring [2,34,49].

As previously mentioned, a high content of phenolic hydroxyl groups in lignin is desired for application in wood adhesives. Additionally, while softwood lignins have been referred to as more reactive, hardwood lignins may still be used in wood adhesives, namely as phenol substitutes [16]. Thus, the reactivity of these samples will be assessed further in a methylolation reaction through the consumption of formaldehyde [20].

**Figure 6:**
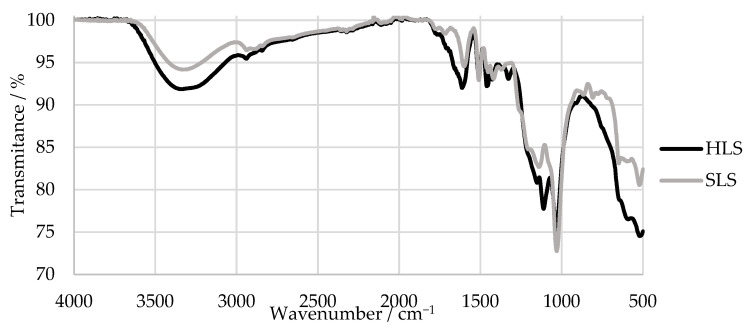
FTIR spectra of the HLS and SLS samples.

An FTIR analysis of the samples was also carried out. The results are shown in Figure 6. The main bands were assigned according to [34,52,53] and are summarized in Table 3.

The FTIR spectra of HLS and SLS both presented a peak characteristic of the LS samples near 1034 cm^−1^ and bands at around 1209 and 644 cm^−1^, which indicate the presence of sulphonic groups [52,54]. Peaks associated with aromatic ring vibrations at around 1613, 1515, and 1426 cm^−1^ were also observed in both samples [52]. It can be concluded that, overall, the samples presented similar spectra.

However, only the SLS sample presents bands typical of G units at 1261, 1140, 865, and 809 cm^−1^. In comparison, the HLS sample presents bands characteristic of S units at 1328 and 1111 cm^−1^ [52]. The missing G bands of the HLS sample are explained by its high S:G ratio (81:19) that was previously reported in other studies [34,51]. In contrast, the SLS sample is expected to be composed almost exclusively of G units [14]. Once again, these results confirm the hardwood and softwood origins of the HLS and SLS samples, respectively.

Lastly, both samples present a band derived from the carbohydrate origin near 1710 cm^−1^. This is due to the fact that these samples still contain a considerable percentage of impurities, as shown in Table 1 by their low LS content [52]. 

### 3.2. Monitoring of the Methylolation Reaction

#### 3.2.1. Off-Line Methods

By following the characterization of the LS samples, their methylolation was studied. The initial quantities of the reactants, as well as their respective concentrations in the reaction medium, are shown in Table 4. The numbers shown in bold are the fixed operating conditions in all trials, as mentioned previously in the methods section.

In order to confirm the validity of in-line monitoring through visible spectroscopy, the reaction was also followed using the previously established off-line methods.

Thus, the reaction progress was followed in terms of the decrease in free formaldehyde content and in the phenolic hydroxyl groups, type I and II. The operating conditions for the SLS sample were a pH of 9.6, a temperature of 60 °C, and a formaldehyde-to-lignin solids mass ratio of 0.17.

**Figure 7:**
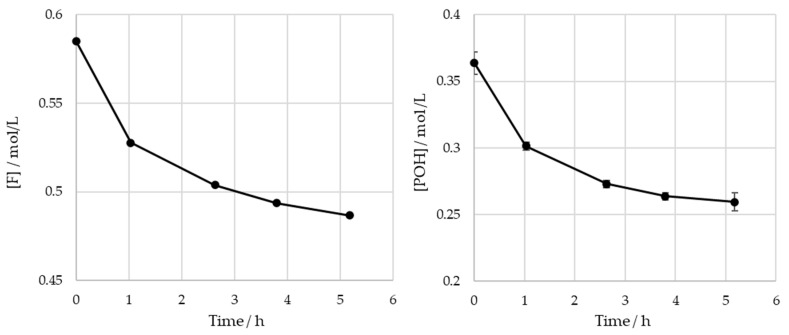
Consumption of formaldehyde and phenolic hydroxyl groups type I and II in the methylolation of SLS at 60 °C.

The obtained results for the SLS sample are shown in Figure 7.

For the operating conditions employed, no significant variation is observed in the content of the phenolic hydroxyl groups after 4 h of reaction (p<0.05). The total consumptions of the formaldehyde and phenolic hydroxyl groups at the end of the reaction are 0.10 mol/L and 0.099 mol/L, respectively. It was concluded that there are no significant differences between these values. As such, under these conditions, the contribution of the Cannizaro reaction to formaldehyde consumption is deemed to be negligible. These findings are also supported by the work of Taverna et al. [26].

A similar analysis was carried out for the HLS sample under three different temperatures: 50, 60, and 70 °C.

**Figure 8:**
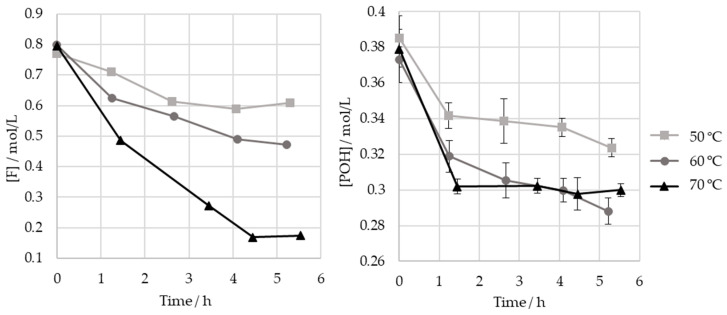
Consumption of formaldehyde and phenolic hydroxyl groups type I and II in the methylolation of HLS at 50, 60, and 70 °C.

As different studies diverge in their selection of the optimum temperatures for the hydroxymethylation of lignin, the effect of the temperature on this reaction was studied here. Thus, the HLS sample was treated at three temperatures: 50, 60, and 70 °C. The results are shown in Figure 8 and Table 5.

As seen in Figure 8, the increase in temperature results in a significant increase in formaldehyde consumption. However, as shown in Table 5, these values do not correlate with the consumption of phenolic groups. It is hypothesized that other components in the sulphite liquor may react with formaldehyde in secondary reactions. This disparity is especially high at 70 °C, possibly indicating that the Cannizzaro reaction may have occurred to a significant extent. Nevertheless, this temperature appears to have accelerated the consumption of the phenolic type I and II groups, which ceased after 1 h (p<0.05).

On the other hand, the final consumption of the phenolic groups after 5 h is lower for 50 °C than for 70 °C and 60 °C (p<0.05). Therefore, in order to minimize the time needed to achieve a high methylolation degree, a temperature of at least 60 °C should be employed under the studied operating conditions.

Regardless of the temperature, most, if not all, of the phenolic groups are consumed in the first hour of the reaction.

To conclude, a temperature of 70 °C may be used, allowing a lower total reaction time. However, this temperature should not be combined with low F/LS rates, as this may lead to competition with undesirable reactions and the Lederer–Manasse reaction [40].

The reaction rate constants of the studied methylolation reactions were estimated from the data alongside the percentage of reactive phenolic groups. These determinations were based on equations 1, 2 and 3 for SLS and 2, 3 and 4 for HLS. For the methylolation of HLS at 70 °C, these values were not determined since the reaction had already ceased after one hour. The results are shown in Table 6.

The percentage of reactive phenolic groups is higher in the SLS sample. This result was expected since hardwood lignin displays more blocked ortho positions due to its predominance in syringyl units. The value for the HLS sample at 60 °C is consistent with the percentage of such units reported for this type of LS in previous studies [34,49,51].

The reaction rate constant for the methylolation of the SLS sample is in accordance with that reported by Peng et al. for another softwood LS sample at the same temperature (1.44 L mol^−1^ h^−1^) [24]. On the other hand, unexpectedly, the methylolation rate constant for HLS, *k*_1_, appears to decrease with the increase in temperature. These results are not consistent with other studies for phenol and lignin methylolation [24,55]. It is admitted that the delay in the off-line methods, in combination with the low number of samples, may have resulted in an inaccurate estimation of this parameter for 60 °C.

#### 3.2.2. In-Line Method–Visible Spectroscopy

**Figure 9:**
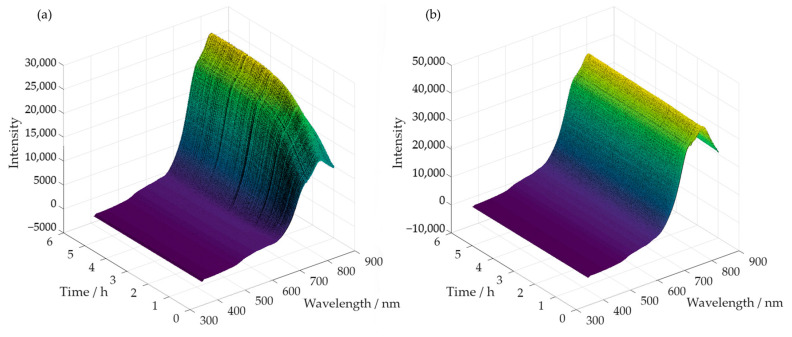
Spectra acquired along the methylolation of SLS (**a**) and HLS (**b**) at 60 °C.

Simultaneously, with the collection of samples, spectra were also acquired throughout the reaction time. Examples of the obtained spectra are shown in Figure 9a,b, for the methylolation at 60 °C of SLS and HLS, respectively.

**Figure 10:**
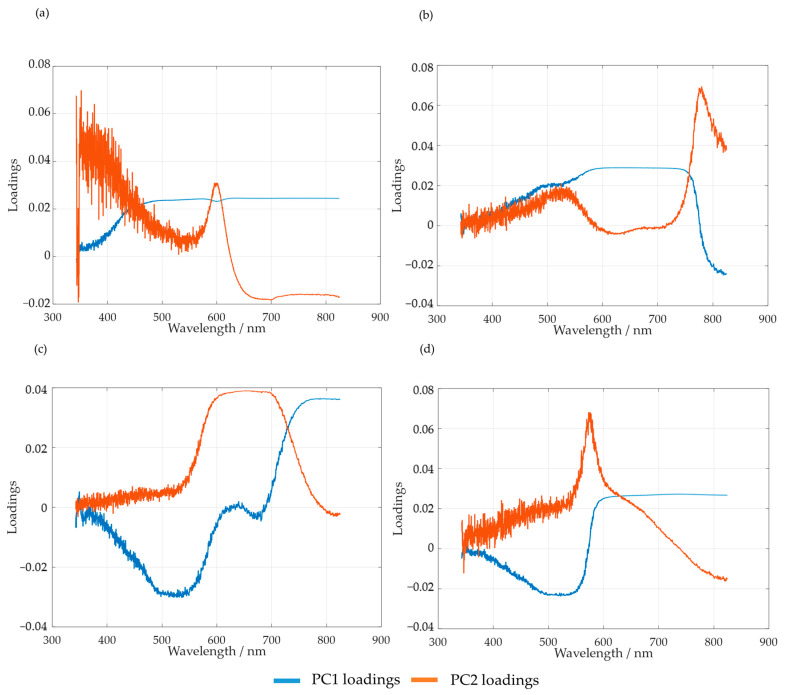
PC1 and PC2 loadings for the methylolation of the SLS sample at 60 °C (**a**) and HLS at 50 °C (**b**), 60 °C (**c**), and 70 °C (**d**).

The spectra obtained were mean-centered, and PCA analysis was carried out. Firstly, the loadings of the first and second principal components, PC1 and PC2, were analyzed. The results obtained for SLS are shown in Figure 10a. For HLS, the results are shown in Figure 10b–d for the temperatures of 50, 60, and 70 °C, respectively.

As suggested by Bernar et al., the loadings of the first principal components were analyzed in order to verify the most relevant spectral bands [25]. The loading analyses for SLS and HLS indicate that the region with the most noise appears to be comprised between 342 and 600 nm. Taking this into account, the selected spectral region for the PCA analyses of both samples was from 600 to 800 nm. 

It should be noted that, for HLS, the first and second loadings appear to switch places when the temperature is raised from 50 to 60 and 70 °C. This is made clear by observing the region between 700 and 800 nm. Simultaneously, the peak at 650 nm also appears to increase with temperature, possibly indicating the increase in secondary reactions.

**Figure 11:**
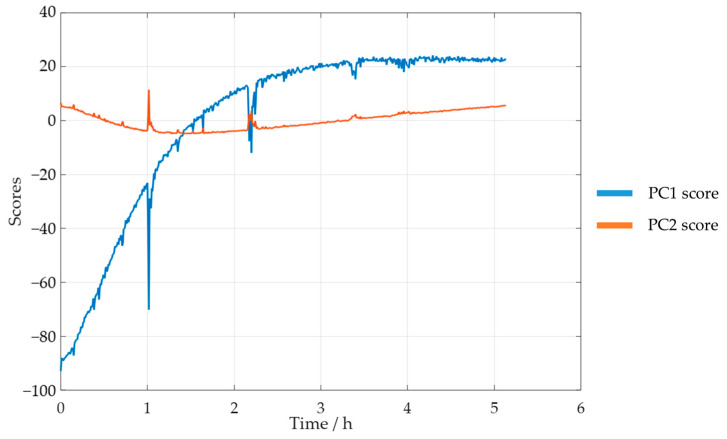
PC1 and PC2 scores for the methylolation of the SLS sample at 60 °C.

Taking these results into account, a new PCA analysis was carried out while only considering the selected spectral region. The reaction progress was verified through the analysis of the scores. The PC1 and PC2 scores for SLS methylolation are shown in Figure 11.

The plot of the PC1 score displays a curve similar to that of the previously determined decay of [POH]. Both curves also stabilized after approximately 4 h. Bernar and co-workers, on the other hand, verified a shift in the PC1 score after 4 h, which was attributed to the end of the lignin methylolation [25]. Thus, these results appear to confirm the possibility of using visible spectroscopy to monitor the reaction. The momentaneous disturbances, observed mainly in the PC1 curve score, were associated with the collection of samples from the reactor for the off-line methods. 

**Figure 12:**
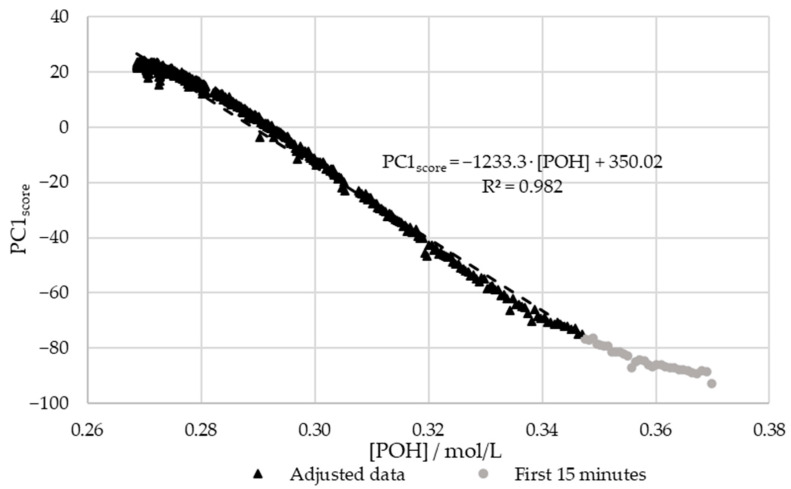
PC1 score vs. the decay of phenolic groups type I and II for the methylolation of the SLS sample at 60 °C.

In order to confirm the relations between the decay of [POH] and the scores of PC1, the results were plotted against each other. For this purpose, the previously determined kinetic parameters were used to plot the [POH] decay curve. The aforementioned extraneous data points were excluded. The results are shown in Figure 12.

Figure 12 shows that there is a satisfactory regression (R^2^ > 0.95) between the in-line and off-line data. Therefore, it can be concluded that visible spectroscopy is an accurate technique for monitoring the methylolation of SLS. It should be noted that the data for the first 15 min of this reaction, shown in gray in Figure 12, was not included in the regression. It is considered that the error in the off-line data is especially high at the beginning of the reaction due to the delay in the analysis. As shown in Figure 7, the error in [*POH*] is most significant at the beginning and end of the reaction. This is also where the deviation between the off-line and in-line data is more significant, as seen in Figure 12. Therefore, the discrepancies are justified by the delay and error of the off-line method.

**Figure 13:**
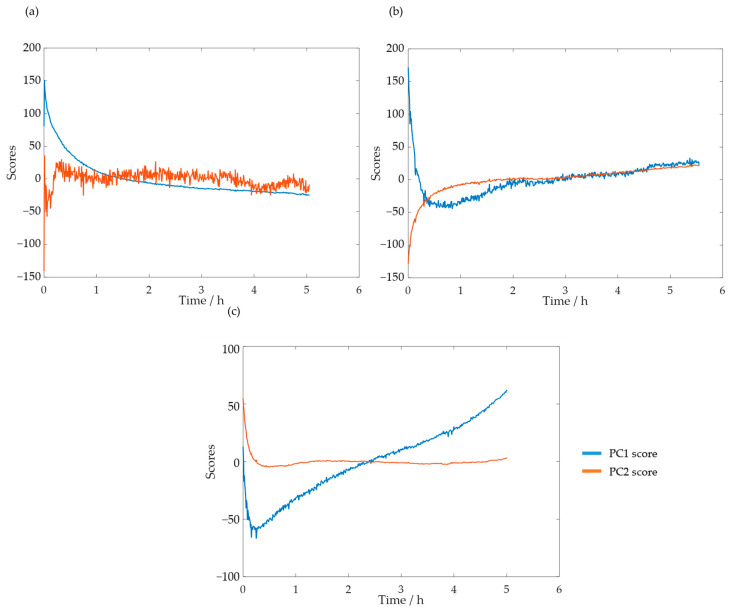
PC1 and PC2 scores for the methylolation of the HLS sample at 50 °C (**a**), 60 °C (**b**), and 70 °C (**c**).

These analyses were also carried out for HLS. The scores for the PC1 and PC2 at the temperatures of 50, 60, and 70 °C are shown in Figure 13a–c, respectively.

As suspected previously through the loading analysis, the scores which contain information on the reaction progress appear to be the first at 50 °C and second at 60 and 70 °C. Notably, at 70 °C, the second score appears to stabilize after just a few minutes. This is in agreement with the off-line results, for which the content of the phenolic groups stabilizes in less than one hour.

**Figure 14:**
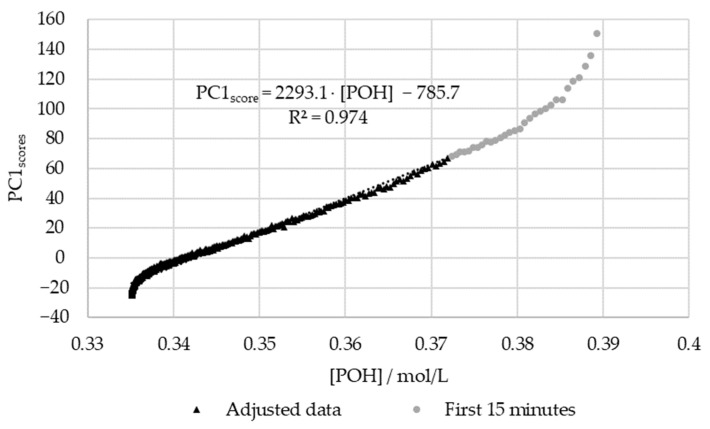
PC1 score vs. the decay of phenolic groups type I and II for the methylolation of the HLS sample at 50 °C.

**Figure 15:**
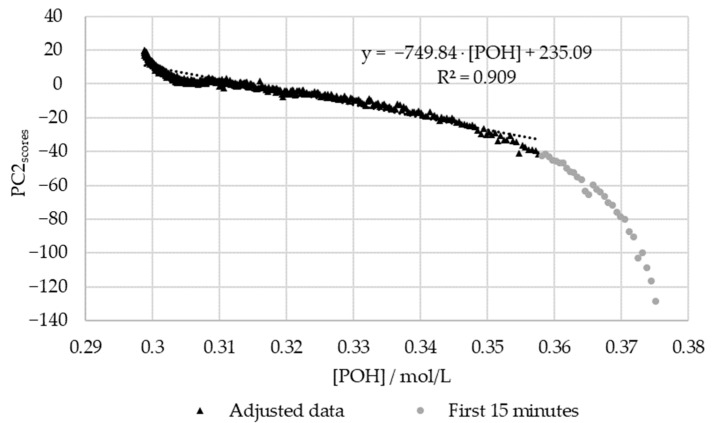
PC2 score vs. the decay of phenolic groups type I and II for the methylolation of the HLS sample at 60 °C.

Once again, in order to confirm the regression between the scores and the off-line data, these were plotted against each other. The results are shown in Figure 14 and Figure 15 for the methylolation at 50 and 60 °C, respectively. This analysis was not carried out for the methylolation at 70 °C due to insufficient data, as previously mentioned.

Figure 15 shows that, once again, the regression between the off-line data and the PC1 scores is high (R^2^ > 0.95). However, for this, the first fifteen minutes were again excluded due to the significant delay in the off-line method. In agreement with the results for SLS, the deviations in the off-line method are especially high at the beginning and end of the reaction. Particularly, in the beginning, the most significant discrepancies are observed within the first 5 min of the reaction ([*POH*] > 0.37 mol/L). At the end of the reaction, the modeled decay of the phenolic groups appears to stabilize, unlike the in-line data. This may be partially explained through the analysis of Figure 8, where the decay of the phenolic groups appears to follow a different reaction rate at the end of the methylolation. This was not accounted for in the second-order model used to fit the data. Further trials would be needed to confirm this behavior.

On the other hand, the off-line data deviated significantly from the PC2 scores at 60 °C (R^2^ < 0.95). This discrepancy is especially high within the first 15 min of the reaction. As previously suspected, more off-line data is needed during the first hour of the reaction in order to accurately determine the reaction rate constant at 60 °C. The delays in the off-line method may have also contributed to these deviations.

Nevertheless, this study proves the potential of visible spectroscopy as an in-line technique for the monitoring and control of the methylolation of lignin samples, even in the presence of secondary reactions.

Future studies should focus on developing partial least square regression models, which would allow the accurate monitoring and study of the methylolation of lignin samples, even at high reaction rates.

## 4. Conclusions

As the low number of reactive sites available in lignin’s aromatic ring and high polydispersity have hindered its application in resin synthesis, methods such as methylolation with formaldehyde have been proposed to improve the reactivity of lignin molecules. However, currently, there is a limited amount of studies on the reaction of formaldehyde and lignin. On the other hand, these studies resort to off-line, time-consuming and destructive methods. Therefore, this study assesses the possibility of using visible spectroscopy as an in-line technique to monitor the methylolation of lignin samples.

Two lignosulphonate (LS) samples from softwood (SLS) and hardwood (HLS) were studied. In the first analysis, off-line methods were employed. The reactions were followed through the consumption of formaldehyde and phenolic hydroxyl groups. SLS proved to be the most reactive since more phenolic hydroxyl groups are consumed under the same operating conditions. Although a large amount of formaldehyde consumption is observed for HLS, this is attributed mostly to undesirable secondary reactions.

Lastly, through the PCA analysis, it was possible to analyze the visible spectra obtained along the reaction time. The obtained scores were related to the previously obtained off-line data. It was concluded that there is a satisfactory coefficient of determination (R^2^ > 0.95) between the two sets of data for the methylolation of the LS samples at 50 and 60 °C. These results prove that visible spectroscopy is a promising technique for the in-line monitoring of lignin methylolation.

Future studies should focus on developing partial least square methods so as to allow for the real-time monitoring of the consumption of phenolic hydroxyl groups and formaldehyde. The accuracy of the in-line methods would also be limited to that of the off-line methods used for the calibration.

Nevertheless, besides allowing for expedite in-line monitoring and control of the industrial process, this would help provide further insights into the reactivity of different lignins and aid its incorporation in resins. For example, the selection of the most promising lignin in a group of samples would be facilitated.

## Figures and Tables

**Table 1:** Physico-chemical characterization results of the HLS and SLS samples.

Parameter	HLS	SLS
Density (g/cm^3^)	1.308	-
Viscosity (cP)	32	-
pH	2.48	-
Dry matter (%)	54.7	95.8
LS content (%)	33.5	72
Carbohydrate content (%)	12.6 ^b^	8.8 ^c^
LS content (%) ^a^	61.2	75.1
Ash content (%) ^a^	12.2	15.7

^a^ on a dry matter basis. ^b^ according to [49]. ^c^ in accordance to the suplier (10 ± 2.0 %) and determined as: Dry matter—LS content—Ash content.

**Table 2:** Content of phenolic hydroxyl groups of the HLS and SLS samples.

Phenolic Hydroxyl Groups (%) on a Dry LS Basis	HLS	SLS
*[OH]_I+II+III+IV_*	2.69	2.23
*[OH]_I+II_*	1.76	1.37
*[OH]_III+IV_*	0.93	0.86

**Table 3:** FTIR absorption band assignments of the SLS and HLS samples [34,49,52,53,54].

Wavenumber (cm^−1^)	Band Origin and Comments	HLS	SLS
3420–3250	O-H stretch (phenolic and aliphatic OH)	3332	3332
3000–2842	C-H stretch in methyl and methylene groups	2940	2938
2850–2840	C-H stretching (OCH_3_)	2844	2843
2000–1650	Several bands from overtones and combinations (substituted benzene rings)	1766	1769
1738–1709	C=O stretch in unconjugated ketones, carbonyls and in ester groups (frequently of carbohydrate origin)	1704	1714
1605–1593	Aromatic skeletal vibrations; C=O stretch	1613	1601
1515–1505	Aromatic skeletal vibrations	1515	1510
1470–1460	C-H deformations (asymmetric in CH_3_ and CH_2_)	1461	1452
1430–1422	Aromatic skeletal vibrations and C-H in-plane deformation	1426	1418
1370–1365	Aliphatic C-H stretch in CH_3_, not in OCH_3_; phenolic OH	1367	1370
1330–1325	S ring and G ring substituted in C_5_	1328	-
1270–1266	G ring; C=O stretch	-	1261
1260–1150	Sulphonic acids	1209	1205
1166	C=O in conjugated ester groups	1151	-
1140	Aromatic C-H in-plane deformation; typical for G units	-	1140
1128–1125	Aromatic C-H in-plane deformation (S units); secondary alcohols; C=O stretch	1111	-
1080–1010	Characteristic LS peak at 1037 cm^−1^; sulfonic acids; deformation of aromatic C-H and C-O in primary alcohols; C=O stretch unconjugated	1034	1030
858–853	C-H out-of-plane in positions 2, 5, and 6 of G units	-	865
832–817	C-H out-of-plane in positions 2, 5, and 6 of G units	-	809
700–600	Sulphonic acids	644	648

**Table 4:** Initial quantities of lignin and formaldehyde.

Sample	Lignin			Formaldehyde		
	Dry Mass/g	*Concentration of Pure Lignin/g/L*	[POH]/mol/L	Mass/g	*Mass Ratio F:LS (Impure)*	[F]/mol/L
**SLS**	95.8	***280***	0.36	16.3	***0.17***	0.59
**HLS**	115	***280***	0.38	19.5	***0.17***	0.79

**Table 5:** Comparison between the consumption of formaldehyde and phenolic hydroxyl groups type I and II in the methylolation of HLS at 50, 60, and 70 °C.

Temperature/°C	50	60	70
F consumed/mol/L	0.16	0.33	0.62
[*POH*] consumed/mol/L	0.062	0.085	0.080

**Table 6:** Reaction rate constants and percentage of reactive phenolic groups for the studied methylolations at 60 or 50 °C.

Sample	SLS	HLS	HLS
Temperature/°C	60	60	50
$k_{1}\left(T\right)$/L mol^−1^ h^−1^	1.53	1.32	2.02
$k_{2}\left(T\right)$/L mol^−1^ h^−1^	-	0.14	0.06
$\%B_{non-reactive}$	71	79	86

## Data Availability

The data presented in this study are available on request from the corresponding author.
